# Stress Proliferation or Stress Relief? Understanding Mothers’ Health during Son’s Incarceration

**DOI:** 10.1177/00221465251330848

**Published:** 2025-04-25

**Authors:** Kristin Turney, Rachel Bauman, MacKenzie A. Christensen, Rebecca Goodsell

**Affiliations:** 1 University of California, Irvine, CA, USA; 2 University of Oregon, Eugene, OR, USA

**Keywords:** criminal legal system, incarceration, intergenerational relationships, stress process perspective

## Abstract

Social stressors proliferate to impair the health of those connected to the person enduring the stressor, but they can simultaneously offer relief from other stressors. Using in-depth interviews with 69 mothers of incarcerated men, we investigate mothers’ descriptions of how the stressor of their adult son’s incarceration impairs their health. First, mothers overwhelmingly describe how the increased instrumental, emotional, and financial responsibilities following their son’s confinement damage their health. Second, despite these increased responsibilities, most mothers simultaneously describe stress relief following their son’s incarceration, which may offset some of their health impairments. Third, these processes are situated in a broader social context, with increased responsibilities most salient when mothers have caregiving relationships with their grandchildren and stress relief most salient when their sons endure cyclical incarceration. These findings, which expand our understanding of the symbiotic harms of incarceration for mothers’ health, highlight the complexity of responses to social stressors.

The rise of the carceral state and the corresponding increase in incarceration throughout the past half-century means that more than one-fifth of U.S. older adults (ages 50 and older) have endured the incarceration of a son or daughter (Enns et al. 2019). The incarceration of an adult child, most commonly an adult son, is concentrated among vulnerable population groups, including people of color who navigate structural racism, those facing economic difficulties, and those residing in underresourced neighborhoods (Kirk and Wakefield 2018). A son’s incarceration, commonly endured in a social context of ongoing adversities, may be particularly challenging for the mental and physical health of older adults, who often simultaneously navigate existing chronic health conditions, caregiving responsibilities, and financial insecurity (Bom et al. 2019; Pittman 2023).

The stress process perspective, which elucidates how stressors are concentrated among vulnerable groups and can generate health impairments, provides a theoretical lens for understanding how a son’s incarceration may deteriorate the health of their mother (Pearlin 1989; Pearlin et al. 1981). Incarceration, a stressor directly endured by one person (Massoglia 2008; Schnittker and John 2007), can have proliferating repercussions for the health of those connected to that person (Pearlin, Aneshensel, and LeBlanc 1997). The health consequences of a son’s incarceration for mothers may be direct because the confinement experience—and the corresponding ambiguous loss—can facilitate worry, distress, and uncertainty (Goldman 2019; Green et al. 2006; Sirois 2020). Incarceration may create additional secondary stressors that, along with the primary stressor of incarceration, either facilitate new health conditions or worsen existing ones.

However, the stress process perspective and its focus on how stressors develop within the embedded social contexts of people’s lives also suggests that the health consequences of a son’s incarceration may be more complicated (Comfort 2008). First, the concept of stress relief highlights how a stressor can alleviate existing stressors (Wheaton 1990). The stressor of a son’s incarceration may assuage other stressors—such as a son’s unhoused status or substance use—and in these instances, offer relief from these existing stressors. Second, the stressor of a son’s incarceration operates within a broader social context, including mothers’ caregiving relationships with their grandchildren and mothers’ exposure to their son’s cyclical incarceration, which may shape responses to the stressor (Pearlin 1989; Pittman 2023; Turney 2017).

In this article, we use in-depth interview data with 69 mothers of incarcerated men, most of who identify as Latina, to examine mothers’ responses to their adult son’s incarceration. First, we find that mothers describe how the primary stressor of a son’s incarceration generates instrumental, emotional, and financial responsibilities that provoke new health conditions and exacerbate existing health conditions. Second, we find that incarceration simultaneously offers relief from other chronic stressors, such as their son’s precarious living conditions and substance use, with mothers reporting health benefits to their son’s relative safety while incarcerated, the ease of comparatively frequent and meaningful communication, and the sobriety often accompanying their confinement. Third, we find that these processes occur in a broader social context, with responsibilities most salient when mothers have caregiving relationships with their son’s children and relief most salient when their sons endure cyclical incarceration. These findings extend existing scholarship on the health repercussions of criminal legal contact by drawing attention to mothers of incarcerated men, an often neglected but critically important group who commonly navigate vicarious incarceration; systematically and rigorously documenting emergent processes of stress proliferation and especially stress relief (and how these processes impair health); and examining how the stressor of a son’s incarceration operates within the broader social context of mothers’ lives.

## Background

### Contextualizing Mothers’ Health

Understanding how stressors—and the social context of stressors—contribute to health among older women is important. Health conditions typically increase throughout the life course, with older adults facing more health challenges than their younger counterparts, and stressors can shape health inequalities that become more pronounced as people age (Deaton and Paxson 1998). The aging process is also often accompanied by altered caregiving responsibilities, changes to financial security, and bereavement of loved ones, which can accelerate precarious health (Bom et al. 2019; Pittman 2023).

Understanding the health of women enduring the stressor of their son’s incarceration is critical because this group is particularly vulnerable to structural disadvantages that can contribute to poor health (Enns et al. 2019). This stressor is concentrated among already vulnerable women, including women of color who endure persistent structural racism and poor women who navigate precarious living conditions and neighborhoods (Kirk and Wakefield 2018). Latinas, for example, are about twice as likely as White women to endure a son’s incarceration by age 50 (Sirois 2020). Repeated exposure to racism, poverty, and other structural disadvantages contributes to weathering, the toll that systemic oppression incurs on the body that contributes to racial-ethnic health inequalities (Geronimus 2023). These weathering processes may be amplified among mothers born outside the United States or undocumented mothers, who face considerable health challenges as they age (Boen and Hummer 2019).

At the same time, Latina mothers commonly adhere to the value of familism, which emphasizes close family relationships and the expectation of prioritizing the family (Campos et al. 2014; Sabogal et al. 1987). Familism is positively associated with health among Latinx individuals (Campos et al. 2014). Specifically, greater family support and close family relationships—critical aspects of familism—alter health behaviors (Katiria Perez and Cruess 2014), promote prosocial behaviors (Schwartz et al. 2010), and contribute to psychological well-being among Latinas (Campos et al. 2014).

### Son’s Incarceration in the Stress Process Perspective

The stress process perspective provides a theoretical lens for understanding the health consequences of a son’s incarceration. This perspective posits that stressors, disproportionately concentrated among vulnerable groups, can have both acute and long-lasting implications for mental and physical health (Pearlin 1989; Pearlin et al. 1981). Accordingly, research documents the deleterious health consequences of incarceration for those enduring confinement (e.g., Massoglia 2008; Schnittker and John 2007) and those connected to them (e.g., Turney 2014). Two aspects of the stress process perspective are particularly relevant to understanding the health consequences of a son’s incarceration.

First, aligned with the life course perspective that highlights the nature of linked lives (Elder 1998), the stress process perspective posits that stressors can proliferate across people, also called “stress contagion” (Barr et al. 2018; Pearlin et al. 1997), and across stressors. Stressors proliferate from the person directly experiencing the stressor to those connected to that person (Pearlin et al. 1997). Consequently, the stressor of incarceration can spill over to the mothers of those enduring confinement. Mothers may experience distress and worry while their child is confined. They may experience concern about the carceral environment, including their loved one’s living conditions, coresidents, and treatment by correctional officers and other staff (Liebling 2011). They may endure uncertainty about the future, including their son’s release date (especially if their son awaits case adjudication), how their son will reintegrate after release, and the prospect of their son’s reincarceration (Turney et al. 2024; Western 2018). Stressors also proliferate to create additional stressors, with a primary stressor leading to secondary stressors that impair health (Pearlin 1989; Pearlin et al. 1997). Indeed, the primary stressor of a son’s incarceration may generate secondary stressors for mothers by forcing mothers to navigate the criminal legal system (Green et al. 2006), provide emotional support to their sons (Condry 2007), and incur financial hardships (Page, Piehowski, and Soss 2019), all of which may impair their health.

Second, and also aligned with the life course perspective (Elder 1998), stressors are situated within a broader social context (Pearlin 1989; Turney 2017). The social context can shape how people respond to stressors, with stressors offering relief from existing chronic stress, such as the ongoing and persistent conditions of the social environment (Wheaton 1990). Research on the criminal legal system shows that though incarceration presents challenges to romantic relationships, it can also facilitate meaningful communication between incarcerated people and their romantic partners and can alleviate worry that romantic partners endure prior to their loved one’s confinement (Comfort 2008; Hood and Gaston 2022; Turanovic, Rodriguez, and Pratt 2012). Other research shows that children’s responses to their father’s incarceration are contingent on the broader social context, with some children enduring more deleterious consequences than others (Turney 2017; also see Wildeman 2010). Therefore, the stressor of a son’s incarceration—which often occurs in a social context necessitating extensive management of their son’s prior criminal legal contact, supporting their son through substance use problems, and uncertainty about their son’s whereabouts or well-being—may provide stress relief for some. Relatedly, aspects of the social context prior to their son’s incarceration may condition mothers’ responses to a son’s incarceration (Goldman 2019).

### Existing Research on the Health Consequences of Son’s Incarceration

The health consequences of incarceration for mothers have received considerably less attention than the health consequences of incarceration for other family members, such as romantic partners or children (Foster and Hagan 2015; Wildeman and Lee 2021). However, several quantitative studies document the deleterious health repercussions of a child’s incarceration (Goldman 2019; Green et al. 2006; Sirois 2020). One study, drawing on a sample of African American mothers in Chicago, demonstrates a positive association between son’s incarceration and mothers’ psychological distress (Green et al. 2006). More recent studies using the National Longitudinal Survey of Youth 1979 provide consistent conclusions, with one study finding a son’s incarceration leads to health limitations among mothers (Sirois 2020) and another study finding a child’s incarceration is negatively associated with mothers’ health (including self-rated health, functional limitations, and depression; Goldman 2019).

These existing studies on the health consequences of a son’s incarceration, which rely on survey data, are not well positioned to understand the complex and potentially countervailing processes that mothers experience after this vicarious incarceration exposure. One article finds that financial difficulties and caregiving burdens explain the relationship between son’s incarceration and mothers’ health (Green et al. 2006), providing suggestive evidence of stress proliferation, but other processes—including countervailing processes of stress relief—may exist (Comfort 2008). In this article, we advance prior research by using interview data, a method especially suited to understanding processes and mechanisms (Bonell, Warren, and Melendez-Torres 2022), to examine how mothers describe their health as a result of their son’s incarceration, paying particular attention to countervailing processes and to how these processes are embedded in the larger social context of mothers’ lives.

## Data And Methods

### Data

To examine stress proliferation and stress relief processes underlying the health consequences of a son’s incarceration, we used data from the Jail and Family Life Study, an in-depth interview study of incarcerated men and their family members (including their mothers) that was designed to examine the consequences of paternal incarceration for children and families. A team of trained graduate students first recruited and interviewed 123 incarcerated men across three Southern California jails. Men were eligible for participation if they had been in jail for at least two months (to limit heterogeneity in experiences), if they had a child under age 18 (because the larger study was focused on the consequences of paternal incarceration), and if they had seen this child in the month prior to their incarceration. We asked men to provide us with contact information for their mothers (and for other family members). We then contacted their mothers to elicit study participation, endeavoring to interview mothers as soon as possible to document mothers’ experiences during their son’s incarceration. Occasionally, men were released before we interviewed their mother, and in these cases, we encouraged mothers to reflect on their son’s confinement (and to distinguish these experiences from when he was released).

The analytic sample included 69 mothers, with interviews occurring between August 2015 and September 2017.^1^ We conducted interviews at a location preferable and comfortable to mothers, usually in their homes, a restaurant, or a park. The interview guide included questions about their son’s incarceration, their relationship with their son, and their mental and physical health.^2^ We focused on “how” questions to elicit narrative information about processes (Tavory 2020). The interview was semistructured; that is, we asked similar questions of all respondents but sometimes varied the question wording and timing to make the interview flow like a conversation. Nearly half of all interviews (n = 33) were conducted in Spanish (with the remainder of the interviews conducted in English). All but one interview was recorded and transcribed verbatim (and the interviewer for the nonrecorded interview wrote extensive field notes after the interview). Interviewers wrote field notes and interview summaries immediately after each interview. Interviews lasted, on average, more than two hours (range = 18–363 minutes). We provided all mothers a $50 Visa gift card for participation.

### Analytic Approach

The analytic approach, drawing on flexible and inductive coding (Deterding and Waters 2021), occurred in three phases.

First, after reading through transcripts and field notes, a team of trained graduate students conducted deductive coding in Dedoose. These deductive codes were primarily descriptive and derived from interview questions. For example, one large descriptive code included “mental health,” which includes all instances where participants describe experiences related to their mental health (including feelings of stress, reports of sadness, and discussions of diagnosed mental health disorders, related to their son’s incarceration and otherwise). We worked as a team to reach consensus on how to apply each deductive code (via coding about 10 transcripts together). The remaining transcripts were coded by one team member and reviewed carefully by another team member (with two coders working with the larger team to resolve discrepancies).

Second, we restricted the data to the deductive codes related to our research questions (including mental health, physical health, coping and social support, economic well-being and support, and incarceration effects). The fourth author of this article, in consultation with the first author, conducted inductive coding of these data, with these inductive codes emerging from the interviews, to fully understand stress proliferation and stress relief processes stemming from the son’s incarceration. Our approach allowed us to inductively and generatively identify insights from the data and then apply these analytic codes in a rigorous and systematic fashion (Deterding and Waters 2021; Knott et al. 2022). Inductive codes included the key themes (e.g., increased instrumental responsibilities) that we describe below. Importantly, this process involved identifying excerpts that mothers described as both emanating from their son’s incarceration and being related to their health.

Third, we wrote analytic memos to ascertain patterns. We first wrote memos for the full sample and while doing so, paid attention to aspects of the social context that appeared to meaningfully shape mothers’ responses to their son’s incarceration. These analytic memos identified two aspects of the social context that were especially important: mothers’ caregiving relationships with their son’s children and mothers’ exposure to their son’s cyclical incarceration. We then systematically read through the inductive codes for mothers with (n = 41) and without (n = 28) caregiving relationships with their son’s children, writing memos that paid attention to similarities and differences across groups. We followed the same systematic procedure for mothers with (n = 51) and without (n = 18) exposure to their son’s cyclical incarceration (defining cyclical incarceration as four or more incarcerations). These memos highlighted the salience of themes across groups (as noted by both their frequency and prominence reported by mothers).

### Sample Description

Table 1 presents descriptive characteristics for the sample. Mothers were 55 years old and had four children, on average. Most mothers identified as women of color, with the majority (77%) identifying as Latina, consistent with the overrepresentation of the Latinx population in Southern California. Another 17% identified as White, 3% identified as Black, and 2% identified as Asian or multiracial. Nearly two-thirds (64%) were foreign-born, and although we did not systematically ask mothers about legal status, 13% disclosed they were undocumented. Nearly three-fifths (57%) were in a marital or cohabiting relationship, and about half (51%) were employed. More than two-fifths (43%) of mothers’ sons were pretrial, awaiting adjudication of their case, and more than half (54%) were serving a sentence (and sometimes a prison sentence, given AB109, California legislation that mandates that individuals sentenced to nonserious, nonviolent, or nonsex offenses serve their sentences in county jails instead of state prisons). More than three-fifths of sons had spent between one and five years of their lives incarcerated, with the remainder split between those incarcerated for less than one year (22%) and those incarcerated for five or more years (16%). Nearly three-quarters of mothers (74%) had sons who had at least four incarceration spells (ranging from overnight stays in jail to lengthy prison sentences).

**Table 1.** Descriptive Characteristics of Mothers.

	Mean (*SD*)/Frequency	%
Mothers’ characteristics		
Age	55 (9)	
Number of children	4 (2)	
Race-ethnicity		
Hispanic/Latina	53	77%
White	12	17%
Black	2	3%
Asian/Pacific Islander	1	1%
Multiracial	1	1%
Educational attainment		
Less than high school	13	19%
High school or GED	8	12%
More than high school	17	25%
Unknown	31	45%
Social class^a^		
Poor	21	30%
Working poor	33	48%
Working class	8	12%
Middle class	7	10%
Relationship status		
Married or cohabiting	39	57%
In a romantic relationship	5	7%
No romantic relationship	21	30%
Unknown	4	6%
Employed	35	51%
Foreign-born	44	64%
Documentation status		
Undocumented	9	13%
Not undocumented	30	43%
Unknown	30	43%
Ever incarcerated	14	20%
Sons’ characteristics^b^		
Sentencing status		
Pretrial	30	43%
Sentenced	37	54%
Unknown	2	3%
Duration of total incarceration		
<1 year	15	22%
1–5 years	43	62%
>5 years	11	16%
Frequency of incarceration		
1–3 times	18	26%
≥4 times	51	74%
*N*	69	

a We considered mothers to be poor if they were unemployed; working poor if they were employed but reported erratic hours, low pay, and few benefits; working class if they worked full-time in positions with some benefits; and middle class if they worked full-time in professional or white-collar careers.

b These characteristics are taken from interviews with sons.

## Results

The findings proceed in three stages. First, we document the mental and physical health challenges that mothers describe as emerging or being exacerbated upon their son’s incarceration. Second, we describe emergent processes that are consistent with stress proliferation, including the overlapping increased instrumental, emotional, and financial responsibilities that mothers report as stemming from their son’s incarceration (and influencing their health), highlighting how these processes are most salient among mothers with caregiving relationships for their son’s children prior to his incarceration. Third, we describe how mothers describe emergent processes that are consistent with stress relief stemming from their son’s incarceration—including relative physical safety, the ease of comparatively frequent and meaningful communication, and the facilitation of sobriety—and we highlight how these processes are especially salient among mothers of frequently incarcerated sons.

### Health Problems Stemming from Son’s Incarceration

Nearly all mothers of incarcerated sons describe mental and/or physical health problems that emerge in response to their son’s incarceration.^3^ Emotional distress, the most common emergent mental health concern (reported by nearly all mothers), includes feelings of sadness, worry, and anger. This distress is often related to the confinement experience, including the possibility of injury from other residents, poor treatment from correctional officers, poor dietary options, and the fear of possible deportation. Emergent physical health concerns, reported less frequently than mental health concerns (although still reported by nearly one-quarter of mothers), most commonly include conditions responsive to stress, such as weight loss, weight gain, and hypertension (Hicken et al. 2014; Tomiyama 2019). Lindsay, a 47-year-old Latina mother, provides an example of a mother who reports health concerns stemming from her son’s incarceration. Lindsay describes concern, guilt, and sadness about whether her son’s basic needs were being met during his 18-month confinement:

I’d get depressed. I’d lose the appetite. . . . It affected me in all regards. It wasn’t easy to sleep comfortably. It wasn’t easy to eat. None of that was easy. It was very hard for me that he was there while I was free. My thoughts were always him, if he were doing all right, what was happening, how is he doing, all of that was very hard for me. I found it very complicated, very depressing not knowing how he was doing.^4^

Most mothers also describe mental and physical health problems that are worsened or prolonged by their son’s incarceration. Consider Endita, a 54-year-old Latina mother. Endita, who first struggled with depression after her husband passed away two years prior to her son’s incarceration, tells us how her feelings of sadness and loneliness worsened after her son’s incarceration. “I get sad when I must go to sleep and I’m alone. That makes me sad, when I have to eat by myself, or when I have to go somewhere by myself, I feel bad. I get very sad,” she says.^5^ Endita’s depressive symptoms affect her physical health, too:

I don’t go out and I don’t eat. I don’t comb my hair. I don’t take a bath. Sometimes it’s two, three days. And the truth is I don’t like feeling that way, so I look for incentives to go out and eat. But sometimes I do get periods of depression, and I lock myself up completely.

Therefore, for mothers like Endita, they attribute their son’s incarceration to worsening of health conditions.

### Stress Proliferation Pathways Following Son’s Incarceration

Consistent with stress proliferation, our analysis shows that the primary stressor of a son’s incarceration generates secondary stressors—including increased instrumental, emotional, and financial responsibilities—with mothers reporting that these secondary stressors generate new health problems or compound existing ones. These processes are reported by both mothers with and without caregiving relationships with their grandchildren prior to their son’s incarceration but are most salient among the three-quarters of mothers with caregiving relationships.

#### Increased instrumental responsibilities

Mothers of incarcerated sons, especially those with caregiving relationships for their son’s children prior to his incarceration, commonly describe how the primary stressor of incarceration generates secondary stressors of increased instrumental responsibilities, describing how both the incarceration and the instrumental responsibilities impaired their mental and physical health. About three-fifths of mothers report incarceration-related instrumental responsibilities in the context of new or worsening health conditions. The most common instrumental responsibilities during the confinement period include providing care for their son’s children and coordinating with the criminal legal system (e.g., managing their son’s case, attending their son’s court dates, facilitating contact between their son and other family members). Other instrumental responsibilities include coordinating with public defenders or other attorneys; providing housing support to their son’s romantic partners; and dispensing advice about getting or staying sober, managing the conditions of confinement, and avoiding reincarceration upon release. Mothers report that these instrumental responsibilities, independently and alongside the emergent increased emotional and financial responsibilities, take a toll on their health.

First, mothers commonly care for their son’s children during his incarceration by serving as primary caregivers or coordinating with primary caregivers, and they describe how these new or accentuated caregiving responsibilities often substantially impair their mental and physical health. Consider Katrijn, a 51-year-old Latina mother. Katrijn’s son, with whom she had a close relationship prior to his incarceration, is serving a two-year jail sentence. Katrijn visits her son frequently, telling us that this instrumental support takes a toll on her emotions. She recounts spending much of their last visit together crying: “The most difficult part for me is when he gets depressed, when he starts crying and tells me he wants to go out. That’s the hardest part for me, to see that I can’t do anything and he gets desperate.”^6^ Katrijn also incurs the responsibility of taking her son’s daughter, who was born during his incarceration, to visit him. These visits require coordination with her granddaughter’s other grandmother, and ultimately, she describes them as being very difficult because both she and her granddaughter could not hug her son.

Similarly, Sandra, a 49-year-old Latina mother, also describes the stress of visitation. She recounts having an anxiety attack that required medical attention on her way home from visiting her son, who had been awaiting trial for five years. She spent the day at the hospital after her throat closed and she could not breathe or speak:

Sometimes I get stressed out. Once I was coming back from visiting him in jail, and I felt something in my chest that I had to go to the hospital. . . . Yes, because that day I heard some [visitors] there say that their sons got five years, others got six years in prison. I think everything I heard these women talk there made me have an anxiety attack. . . . The doctor told me to relax, to stop thinking so many things, because that was affecting me.^7^

Therefore, mothers like Katrijn and Sandra describe instrumental responsibilities—such as caregiving and visitation—arising from the incarceration in a manner that is consistent with stress proliferation.

Additionally, mothers report increased interactions with the criminal legal system following their son’s incarceration, which they describe as creating or exacerbating their health challenges. Weezie, a 61-year-old Latina mother, describes the instrumental (and financial) responsibilities she incurred during her son’s multiple incarcerations, commonly navigating her son’s paperwork and paying his restitutions. She also facilitates contact between her son and other family members, bringing his nieces and grandmother to visit him in jail even though these visits cause her considerable distress. She says, “I’d get very emotional . . . I’d stress myself out knowing that, okay, so here comes Sunday, I’m gonna go see him, and I’d stress myself out. And then I’d have to talk myself out of it and don’t get emotional.” Weezie also describes living in a constant state of fatigue after incurring these obligations.

Similarly, Regal, a 44-year-old Latina mother, describes how she experienced immediate stress and new depressive symptoms when her son became incarcerated. Regal worked to get custody rights of her grandchild, crying throughout the interview as she recounted this process. With her son incarcerated, Regal stepped in as an informal primary caregiver for her granddaughter alongside her granddaughter’s mother. Regal describes picking up her granddaughter from the mother one day to find that “She was dirty. Her hair was all cut up. She looked like she had been left for hours just to do whatever she wanted.” Regal took her granddaughter to the hospital, prompting her to fight for her granddaughter’s custody while her son was incarcerated: “Long story short, we battled. Spent I would say about $8,000 on an attorney, trying to get her back.” With her son in jail and unable to care for his daughter himself, Regal took on the responsibility to struggle in court: “We went through hell trying to get [her].” The custody battle ended in favor of her granddaughter’s mother, breaking Regal’s and her son’s hearts and exacerbating her depression. Therefore, for Weezie, Regal, and other mothers, instrumental responsibilities, such as increased interactions with the criminal legal system, are consistent with a stress proliferation pathway.

#### Increased emotional responsibilities

Most mothers, especially those with caregiving relationships for their son’s children prior to his incarceration, endure increased emotional responsibilities as they provide critical emotional support to their families. These increased emotional responsibilities include the emotional labor of supporting their incarcerated son, caring for their grandchildren, and managing their son’s relationship with his children. These compounding emotional responsibilities often facilitate new or worsening mental health concerns, including increased symptoms of depression and anxiety. They also manifest physically; mothers link these increased responsibilities to their worsening physical health, including high blood pressure, insomnia, and weight loss. About three-fourths of mothers report incarceration-related emotional responsibilities in the context of new or worsening health conditions.

The mental health consequences of increased emotional responsibilities following a son’s incarceration is illustrated by Lola, a 49-year-old White mother. Lola, given her own incarceration history, is acutely aware of the realities of the carceral system, an insight that only exacerbates her sadness and worry. Lola tells us that the emotional labor of supporting her grandchildren, whom she cares for on weekends, is emotionally taxing: “Watching my grandkids cry over their parents, that stresses me the hell out. . . . And, just not having him there, I mean, it really is a huge void in their life. It impacts them severely. And it is sad, very heartbreaking.”

Kaylee, a 49-year-old Latina mother, also serves as a caregiver for her grandchildren during her son’s incarceration. Kaylee tells us that she struggles with depression, with her symptoms exacerbated by her son’s recent carceral spell. She explains that the hardest part of her son being in jail is “not being able to help him . . . I feel impotent. I can’t even put it into words. I try not to feel more pain.”^8^ Alongside feeling helpless, Kaylee tells us that her mental health is strained by the emotional support she provides to his children. Kaylee reports that her distress regarding her son and grandchildren is heightened by her worry that her son will be deported following his jail stay. She explains, “His children need him . . . now that Father’s Day is coming, [her granddaughter] says she won’t be able to hug her dad because he is in jail, that she can’t give her dad a gift. All of that hurts and it’s so sad.” For mothers like Lola and Kaylee, the primary stressor of their son’s incarceration creates emotional responsibilities that impair their mental health and exacerbate existing conditions.

Mothers also describe the physical health consequences of emotional responsibilities stemming from their son’s incarceration. Marsha, a 48-year-old Latina mother who describes having a very close relationship with her son, visits him in jail about every other week to provide him emotional support. She describes these visits: “It’s so sad. It’s a sad place to be in. It’s a sad place for anyone to wanna go in there. The only reason I do it is to maintain his morality.” Marsha explains that the emotional responsibilities of supporting her incarcerated son, his youngest daughter (whom she cares for several days a week), and her own young daughter became almost too much to bear. She describes how the stress of these emotional responsibilities increased her blood pressure and almost caused a stroke:

I was stressing really bad because I had my granddaughter . . . and I didn’t know how to control that. My blood pressure went through the roof. And I’m like, okay, this is not working out. Since I caught myself screaming at my granddaughter and that’s not like me. . . . And sure enough, it was 159. That’s very high. It’s stroke pressure.

Similarly, Lili, a 54-year-old Latina mother, describes the physical health consequences of the increased emotional responsibilities of supporting her son, his partner, and her two grandchildren. The incarceration of Lili’s son takes a considerable toll. Lili explains that she is chronically worried about her grandchildren’s relationships with their father. Recounting the last time she took her grandson to the jail to see his father, Lili describes the weight of emotionally managing this relationship: “I put the baby in the car seat. And all the way home I cried because I had to let it out.” This stress about her grandson’s well-being also manifested itself physically; she describes how her worry led to a 35-pound weight loss: “I take a blood pressure pill in the morning, one in the evening. But I’ve lost this tremendous amount of weight now compared to what I was.” Therefore, for mothers like Marsha and Lili, the primary stressor of their son’s incarceration generates secondary stressors of emotional responsibilities, and they describe both the primary and secondary stressors as impairing their physical health.

#### Increased financial responsibilities

The primary stressor of a child’s incarceration leads to secondary stressors of increased financial responsibilities, especially among mothers with caregiving responsibilities for their son’s children prior to his incarceration, and mothers describe the mental and physical toll of economically supporting their son and their families during his incarceration. Mothers describe facing accumulating financial responsibilities, including the direct costs of the criminal legal system (e.g., legal fees, attorney, commissary accounts, phone calls) and the indirect costs of covering their son’s ongoing financial obligations during his confinement and financially supporting their son’s children and, at times, mothers of their children. Many mothers describe the financial burden of their son’s incarceration as worsening their health as they sacrifice their own well-being to financially support their son and their son’s families during this time. About half of mothers report increased financial responsibilities in the context of new or worsening health conditions.

Kaylee, introduced earlier, incurred financial responsibilities following her son’s incarceration, responsibilities she describes as causing considerable stress and impairing her health. Kaylee reports health conditions including high blood pressure, diabetes, a hernia, insomnia, and depression. She reports that some of these health conditions—including high blood pressure, diabetes, and depression—have been exacerbated by her son’s incarceration, but others, particularly insomnia, emerged since he was incarcerated three months ago. She describes how the financial responsibilities stemming from her son’s incarceration (e.g., putting money on his commissary account, paying for phone calls) facilitated sleepless nights and feelings of helplessness. She visits her son weekly (forgoing phone calls with him due to their cost), and she often brings his young children, for whom she has caregiving responsibilities, with her. She puts between $20 and $30 weekly on his commissary account. She describes her son’s incarceration as creating substantial economic hardship for her family, contributing to her stress: “When the bills come and there isn’t enough money. When I open the fridge and there isn’t money. When I need to go to the doctor and there isn’t enough money. When I know that the kids aren’t sleeping or eating well. It makes me feel stressed, depressed.”^9^ She says that her sadness disappears when she envisions her son getting out of jail: “I know that my depression is going to go away once he is out and I see him with his kids. Happy.”

Mae, a 53-year-old White mother, also describes the health consequences of financially supporting her son’s incarceration. Reflecting on her son’s most recent incarceration experience, Mae explains, “It was hard, it hurt. . . . It takes up your time. It takes up your money. It’s hard. And then when you don’t have the money for a lawyer, it’s even worse. You just have to sit there and ride it out.” Mae describes her financial situation as precarious, explaining that there are times when she cannot afford food, but she continues to support her son regardless of the consequences for her own health. When asked about what makes her worry, Mae explains,

Not being able to pay my bills, when I start running out of groceries, when I have no gas in the car to get to work. [My son] really makes me worried. I think he’s my top worry. He’s my top worry. Everything else I’ll let go out the door.

Therefore, for mothers like Kaylee and Mae, their impaired mental health stems from the primary stressor of their son’s incarceration and the secondary, proliferating stress of the corresponding increased financial responsibilities.

In addition to the financial costs of their son’s incarceration, mothers also describe the mental and physical toll stemming from the financial responsibilities of caring for grandchildren. Consider Rosie, a 59-year-old Latina mother, who describes chronic fatigue in response to her long work hours, a necessity to keep up with the mounting costs of caring for her grandchildren. Her son’s incarceration worsened her physical tiredness as she balanced paid work, attending her son’s court dates, and her caregiving responsibilities for her other children and grandchildren. “I just work to pay rent, the phone, the bills, and to survive . . . I come home, sleep two to three hours. Then I have to go to court. Then I have to go see [my grandchildren]. I go to work and I’m falling asleep,” she says.^10^ A son’s incarceration facilitates increased financial responsibilities for many mothers, especially those caring for their son’s children like Rosie, which creates new health conditions or exacerbates existing ones.

### Stress Relief Pathways Following Son’s Incarceration

A son’s incarceration is stressful, and nearly all mothers of incarcerated sons report secondary proliferating stressors of increased instrumental, emotional, and financial responsibilities. Nearly all mothers also report that both the primary stressor of incarceration and these secondary stressors impair their mental and physical health. However, although the incarceration experience is fraught with proliferating stressors, all but two mothers report some form of stress relief stemming from their son’s incarceration, a sentiment that is especially salient among the nearly three-quarters of mothers whose sons have endured frequent carceral spells.^11^ The most common (and occasionally overlapping) domains of stress relief include reports that their son is physically safer incarcerated than in the community, the ease of frequent and meaningful communication during the carceral period, and the facilitation of sobriety accompanying incarceration. Mothers also occasionally report stress relief from witnessing transformations in their son’s behavior, their son’s participation in programming (e.g., GED classes, parenting classes, Alcoholics Anonymous classes), and their son’s structured daily activities while incarcerated. This stress relief and its corresponding health enhancements likely dampen some of the deleterious health repercussions endured by mothers.

#### Physical safety during incarceration

Many mothers, especially those of sons who have endured cyclical incarceration, describe how their sons are physically safer while incarcerated than in the community. This theme is the most prevalent form of stress relief among mothers (reported by two-thirds of participants). Mothers describe how incarceration protects their sons from the physical harm they experience on the street and from friends who bring them into dangerous situations, which offers mothers a sense of relief. The incarceration period brings worries about safety, with mothers acutely aware of the unhealthy living conditions and exposure to violence that accompanies incarceration, but some mothers note that the conditions of confinement cannot be worse than the conditions of the street.

Consider Maria, a 48-year-old Latina mother. Her son has been incarcerated for nine months, his fifth or sixth jail stay, and she attributes some of her chronic health conditions (including diabetes, arthritis, and depression) to his incarceration. She says,

I get a lot of anxiety. Because I get depressed. I don’t want to cry. I don’t want to feel anything. . . . That anxiety ends up with my bones. They say that’s why I have arthritis. I don’t want to worry but it makes me sad. It takes over my soul.^12^

Maria recognizes that confinement comes with safety concerns, but she also describes relief accompanying his incarceration: “Of course, these are terrible things there [in jail], but it is easier . . . to know that he is in the hands of justice than that he is on the streets.” She explains that confinement in jail is a safer environment for her son because “on the streets it is too dangerous. There are a lot of dirty people that want to get you even dirtier.” Maria tells us that she is hopeful about her son’s incarceration, explaining,

I hope that all of this helps him. I hope that he comes out, but in a different way. If not, it’s better that he stays here. Here, it is a headache to have him. I don’t sleep because I’m thinking: Where is my son? What is my son doing? When are they going to bring me bad news? I shudder to think about these things.

Maria, like many mothers, reports both hardship and relief stemming from her son’s incarceration. These mothers describe how the primary stressor of incarceration creates and exacerbates health problems, but it is likely these deleterious health consequences would be even greater without the relief simultaneously provided by the incarceration.

Weezie, introduced earlier, provides another example of the stress relief experienced by mothers of incarcerated sons. Like many mothers, Weezie describes the exacerbation of existing mental health problems (including worry and sadness) upon her son’s incarceration. However, Weezie also tells us she is physically safer from her son when he is incarcerated, emphasizing the comfort she feels—and corresponding better sleep she has—when her son in jail. Her son began churning through jail over the past couple of years, usually for a few days to a month at a time, and she reports conflicting emotions about his confinement. She says the following about his current jail stay: “Easy. I don’t like seeing him in there. I mean, no, but it’s peaceful for me.” She continues, telling us that she does not worry about his safety while he is confined:

You know, what he’s doing is, he gonna get shot out on the streets, is he gonna kill himself doing his crap . . . I’m gonna get this knock on the door, “We just picked up your kid, he’s dead.” Or is he gonna come knocking at my door and start breaking my windows? Is he gonna hide out some place around my house? I’m at peace.

Like Weezie, mothers commonly describe how their son’s incarceration relieves them from the stress of not knowing his whereabouts. They report uncertainty about their son’s whereabouts as generating worry about his well-being. They also describe anticipation of hearing bad news from the police that their son has gotten into trouble, been arrested, or died. When they do not know where their sons are, mothers spend time and energy tracking down their sons (via internet searches of jail rosters, calling hospitals, or contacting their son’s friends). Consider Caisa, for example, a 60-year-old Latina mother who has endured her son’s cyclical incarceration. Unlike most mothers, Caisa does not report new or worsening health problems stemming from her son’s incarceration. Instead, Caisa’s health problems emerge when her son is not incarcerated or when she anticipates her son being released. She reports difficulty sleeping when he is not incarcerated because she is anxious about his whereabouts and his potential reincarceration. She says:

It’s ugly but not difficult for him to be in jail. I am more relaxed when he was in [jail] . . . I am more relaxed because I know where he is. It’s hard when he is in the street. That’s very hard and sad. I couldn’t sleep well because I was thinking whether he was alive or not, whether he was eating or not. Then when we eat, he wasn’t there. And with him in jail, there is calm. I am sad but calm.^13^

Caisa is one of only four mothers in our sample who report stress relief without reporting stress proliferation (i.e., increased responsibilities they report as contributing negatively to their health), but her reports of stress relief are common among mothers.

#### Ease of frequent and meaningful communication

More than one-third of mothers describe stress relief stemming from more frequent and effective communication with their incarcerated sons, a pattern especially salient when their sons endure cyclical incarceration. Many mothers emphasize how their son’s incarceration helped facilitate regular communication through visits, calls, and letters. They describe how their sons are often more accessible while incarcerated than when not incarcerated.^14^

Lynn, a 66-year-old White mother, provides an example of a woman who, in the face of considerable stress stemming from her son’s cyclical incarceration, also reports stress relief that ameliorates some of the health consequences of his confinement. “I’m just totally depressed over the fact he’s back in [jail], but I understand that that’s his path that he’s chosen and there’s nothing I can do about it.” She tells us about the financial resources she has contributed to his incarceration over the years—in the form of providing postage stamps, putting money on his commissary account, and paying for collect calls—illustrating a pathway of stress proliferation. “I would hate to stop and think of how much money I’ve put on [my son’s] books and boxes I’ve sent him. It would just be overwhelming. I don’t even want to think about it.” Despite these increased financial responsibilities, Lynn describes how the increased communication during his incarceration (along with the knowledge of his whereabouts) has strengthened their relationship. “I don’t like the fact that he’s in [jail]. But I’m talking to him and he writes on a regular basis and, you know, he’s very caring, he always has been. So that’s not a bad thing.” Similarly, Marisol, a 36-year-old Latina mother who is recovering from a stroke, tells us that although her son’s cyclical incarceration created considerable distress and worry, it has also strengthened their relationship. “Now he talks more with me. Now he tells me that he wants to do things right, that he wants me to help [his child’s mother] to take care of [his son], like a family. He wants to improve.”^15^ Mothers like Lynn and Marisol describe stress relief from more frequent and meaningful communication with their incarcerated sons, which likely helps offset some of the stress proliferation pathways they also experience.

#### Sobriety accompanying incarceration

Finally, in addition to the physical safety and meaningful conversation described previously, mothers describe stress relief during their son’s incarceration because it facilitates sobriety (and relatedly, productive reflection and transformation). This is reported by about one-third of mothers. Incarceration, and the removal of sons from contexts where drug and alcohol use are abundant, can provide sons with an environment for getting sober. Mothers describe how sobriety makes their sons healthier by both improving their son’s health and strengthening their son’s relationships. This facilitates stress relief among mothers, which may partially mute the deleterious health consequences of having an incarcerated son.

For example, Max, a 48-year-old White mother, describes the relief that she feels when her son is incarcerated, off the streets, and without access to substances. Max describes incurring considerable responsibilities in her son’s repeated absences, including caring for his two young children, which generates stress and worry for her. This stress and worry, though, is partially offset by the relief accompanying his incarceration. She tells us that losing her son to heroin is a much greater stressor than his incarceration, describing a “weight’s lifted off my shoulders.” She says,

At least when he goes to jail, I know that he’s safe. . . . Because I know that heroin’s gonna kill him. It’s going to kill him unless he gets off it. And the only time he can get off it is when he’s in there. So when he gets picked up, it sucks. But I’m relieved at the same time knowing that.

She also tells us that her son has gained weight during his most recent incarceration, which she views as a positive transformation.

Similarly, Kaylee, introduced previously, describes stress relief accompanying her son’s incarceration. She tells us how the financial responsibilities stemming from her son’s cyclical incarceration impair her health and that on balance, her health suffers from her son’s incarceration. She also tells us, though, that her son’s most recent incarceration curbed his substance use:

I wasn’t upset. I thanked God that he went to jail. I thank the police for catching him because that way he stopped doing the bad things he was doing before. He can’t do those things inside jail. He was very skinny because he didn’t eat because of drugs and his face looked ill. He didn’t think anymore. So, I wasn’t upset, I told him that I rather see him inside than outside in the state that he was in.^16^

Therefore, although Kaylee’s health undoubtedly worsened during her son’s incarceration, as described previously, the stress relief accompanying his incarceration likely tempers these health impairments.

## Discussion

The stress process perspective highlights that the consequences of social stressors proliferate to the loved ones of those directly enduring the stressor (Pearlin et al. 1997). The stress process perspective also suggests that the consequences of stressors are dependent on the social context and that in some cases, these repercussions offer relief from other stressors (Pearlin 1989; Wheaton 1990). We pay attention to these complexities by drawing on in-depth interview data with 69 mothers of incarcerated men, most of whom identify as Latina, providing a systematic accounting of the processes through which a son’s incarceration impairs their mothers’ health. A child’s incarceration is a common stressor, affecting more than one-fifth of older U.S. adults (Enns et al. 2019; Sirois 2020), but existing research that examines the health repercussions of this stressor for mothers has yet to identify the complex and countervailing processes stemming from vicarious incarceration exposure (Goldman 2019; Green et al. 2006; Sirois 2020). This is a particularly important oversight given that a son’s incarceration is commonly endured in a social context of ongoing adversities (Kirk and Wakefield 2018) and caregiving responsibilities for other children and grandchildren (Pittman 2023). Our data reveal three key findings.

First, we find evidence consistent with stress proliferation. Mothers overwhelmingly report that the primary stressor of incarceration increases instrumental, emotional, and financial responsibilities and that they attribute these increased responsibilities as generating new health conditions and exacerbating existing health conditions. This aligns with the stress process perspective, which posits that an event endured by one person can impair the health of those connected to them (Pearlin 1989; Pearlin et al. 1997). This is consistent with survey research documenting the repercussions of incarceration for mothers’ health (Goldman 2019; Green et al. 2006; Sirois 2020) but extends this research with rich narrative data that systematically and rigorously highlight the processes underlying this relationship. A son’s incarceration provokes considerable burdens for mothers that they describe as impairing their health. Mothers report instrumental responsibilities, such as providing care for their son’s children and coordinating with the criminal legal system; emotional responsibilities, such as supporting their incarcerated son, providing emotional care for their grandchildren, and managing their son’s relationship with his children; and financial responsibilities, such as the costs of criminal legal contact and economically supporting their son and his family during the carceral period, consistent with other research on the consequences of family member incarceration (Condry 2007; Green et al. 2006; Page et al. 2019). Mothers overwhelmingly describe the stressor of the incarceration period, likely because our interview questions focused on that experience, but some increased responsibilities may emanate from criminal legal contact more generally (e.g., mothers may help their sons cover the fines and fees that commonly accompany an arrest without incarceration).

Second, aligned with the stress process perspective’s proposition that stressors develop within the social contexts of people’s lives, we find that the stressor of a son’s incarceration can offer relief from existing chronic stressors (Hood and Gaston 2022; Wheaton 1990). Most mothers disclose some form of stress relief accompanying their son’s incarceration, with the most common forms of stress relief including reports that their son is safer incarcerated than in the community, the ease of frequent and meaningful communication during the carceral period, and the facilitation of sobriety accompanying incarceration. Mothers usually describe stress relief in the context of simultaneously enduring considerable increased responsibilities that impair their health, suggesting that stress relief offsets some of the deleterious health consequences of their son’s incarceration. Our qualitative data do not allow us to precisely disentangle the unique—and potentially countervailing—causal contributions of stress proliferation and stress relief pathways to mothers’ health, but this is an important direction for future survey research on stress, the criminal legal system, and health.

Third, and similarly aligned with the stress process perspective’s proposition that stressors develop within the social contexts of people’s lives, mothers report processes of stress proliferation (i.e., the increased responsibilities they attribute as impairing their health) and stress relief differentially across social contexts (Pearlin 1989). Stress proliferation is most salient when mothers had caregiving relationships for their son’s children prior to their incarceration (Goldman 2019). These mothers with existing caregiving relationships were often firmly integrated into the lives of their grandchildren, often residing with their son and his children prior to his incarceration, so their son’s absence created additional caregiving burdens (e.g., facilitating visitation and communication between son and his children, increased time spent watching grandchildren). Prior caregiving responsibilities compound the stressor of a son’s incarceration, and these mothers are especially likely to endure instrumental, emotional, and financial responsibilities that take a toll on their health. Stress relief is most salient among mothers of sons who had endured cyclical incarceration, and these mothers commonly withstand other chronic stressors (e.g., son’s precarious housing, substance use). This finding is consistent with the idea that chronic stressors associated with incarceration, as opposed to incarceration itself, at least partially explain the health consequences of a son’s incarceration (Sirois 2020).

### Limitations

The analyses provide the first systematic accounting of the processes through which the primary stressor of a son’s incarceration creates secondary stressors—and simultaneously offers relief—for their mothers, but sample characteristics should be kept in mind when interpreting the results.

First, our analysis considers health consequences for mothers (and not fathers) of incarcerated sons (and not daughters). Men and women have different responses to stress (Kessler and McLeod 1984), and therefore, the processes linking son’s incarceration to their fathers’ health may differ from those of mothers. Similarly, stress proliferation and relief pathways may be especially pronounced among mothers of incarcerated daughters given that incarcerated women are more commonly children’s caregivers (U.S. Department of Justice 2000) and incarcerated women are especially disadvantaged (Heimer, Malone, and De Coster 2023). Examining sex differences in both exposure and responses to stressors is an important direction for future research.

Second, most women in our sample identify as Latina. This advances research on the health consequences of vicarious criminal legal contact among this group, and furthermore, our supplemental analyses show no meaningful differences in processes between Latina and non-Latina women. However, our findings might be different if our sample included more Black women, an especially important group to understand given their overrepresentation in exposure to vicarious criminal legal contact and their unique health vulnerabilities (Enns et al. 2019).

Third, the broader context of sons’ criminal legal contact—besides the frequency of their incarceration—may shape mothers’ responses to the incarceration. For example, the anticipatory stress surrounding pretrial detention and the corresponding uncertainty about when and how their son will be released may shape mothers’ health (Turney et al. 2024). Relatedly, the context of the criminal legal system in California may shape mothers’ responses, and the processes identified may vary if the sample were recruited from different states. Legislation in California mandates that some people serve their prison sentences in local jails, facilities that are commonly closer to families (compared to prisons), which may mean more frequent visitation (that could lead to different stress proliferation and relief pathways). Similarly, state variation in the conditions of confinement could shape mothers’ reports of stress relief (Wildeman, Fitzpatrick, and Goldman 2018).

## Conclusion

This article forwards scholarship on criminal legal contact, health, and inequality by using in-depth interview data, data uniquely positioned to understand processes, to examine how a commonly experienced stressor can create and worsen health conditions among mothers. First, we find evidence consistent with stress proliferation, with the primary stressor of a son’s incarceration facilitating secondary stressors of increased instrumental, emotional, and financial responsibilities, all of which mothers describe as impairing their mental and physical health. Second, consistent with the idea that stress operates in a social context, we find evidence of countervailing processes. Mothers describe their son’s incarceration as offering relief from other stressors (even if this relief is usually accompanied by stress proliferation pathways). Third, stress proliferation processes are most salient when mothers have caregiving relationships for their son’s children prior to his incarceration, and stress relief processes are most salient when mothers have sons who endure cyclical incarceration. We advance research on the consequences of family member incarceration by focusing on how children’s criminal legal contact can be consequential for their parents, as opposed to the more commonly considered intergenerational consequences of parental incarceration for children (Foster and Hagan 2015); systematically identifying processes underlying the health consequences of vicarious incarceration exposure; and describing how stressors develop in a social context that involves countervailing and contingent processes. These findings also expand our understanding of the repercussions of incarceration more broadly, showing how the criminal justice system in the United States has cross-generational consequences for families.

## Notes

1. One mother was the legal guardian of the incarcerated person. Another mother had two sons enrolled in the study. Reasons for not having a mother interview corresponding to each son interview include mother could not be located (n = 21), mother did not respond to the invitation (n = 15), mother is deceased (n = 13), mother declined participation (n = 3), and mother does not speak English or Spanish (n = 1).

2. Importantly, the goal was not to diagnose women with specific health problems, such as major depressive disorder or high blood pressure, but instead to elicit narrative health information. Examples of interview questions include the following: “How has this stay in jail been for you?”; “What have been the most difficult things about him being locked up?”; and “What kinds of things make you feel worried?”

3. Although most mothers reported new or exacerbated health conditions stemming from their son’s incarceration, a handful of mothers (n = 3) did not report worsening health upon their son’s incarceration. Mothers who did not report worsening health had no recent contact with their son, lived in another state or country, and endured considerable adversities themselves (e.g., financial precarity, drug use, homelessness) that overshadowed their son’s incarceration.

4. Interview was translated from Spanish.

5. Interview was translated from Spanish.

6. Interview was translated from Spanish.

7. Interview was translated from Spanish.

8. Interview was translated from Spanish.

9. Interview was translated from Spanish.

10. Interview was translated from Spanish.

11. Most mothers (n = 63) report both stress proliferation (i.e., increased responsibilities they report as contributing negatively to their health) and stress relief. It is less common for mothers to report only stress proliferation (n = 2) or only stress relief (n = 4).

12. Interview was translated from Spanish.

13. Interview was translated from Spanish.

14. Although mothers commonly report stress relief stemming from this increased communication, a few mothers report increased challenges to communicating with their son, especially because they could not contact their sons whenever they wanted or because they struggled to have meaningful conversations with their incarcerated sons.

15. Interview was translated from Spanish.

16. Interview was translated from Spanish.
